# Advanced Prediction and Analysis of Delamination Failure in Graphite-Reinforced Epoxy Composites Using VCCT-Based Finite Element Modelling Techniques

**DOI:** 10.3390/polym17060771

**Published:** 2025-03-14

**Authors:** Ahmed F. Mohamed, Mohammed Y. Abdellah, Mohamed K. Hassan, Ahmed H. Backar

**Affiliations:** 1 Industrial Engineering Department, College of Engineering and Architecture, Umm Al-Qura University, P.O. Box 5555, Makkah 21955, Saudi Arabia; afmohamed@uqu.edu.sa; 2 Mechanical Engineering Department, Faculty of Engineering, South Valley University, Qena 83523, Egypt; 3 Mechanical Engineering Department, College of Engineering, Alasala Colleges, Dammam 31483, Saudi Arabia; 4 Mechanical Engineering Department, College of Engineering and Architecture, Umm Al-Qura University, P.O. Box 5555, Makkah 21955, Saudi Arabia; mkibrahiem@uqu.edu.sa; 5 Production Engineering Department, Faculty of Engineering, Alexandria University, Alexandria 21544, Egypt; ahmed.backar@alexu.edu.eg

**Keywords:** virtual crack closure technique, surface release energy, graphite reinforcement, double cantilever beam

## Abstract

The applications of graphite-reinforced composite laminates have gained significant importance since the last century and remain a highly attractive field due to their widespread and versatile applications. Among the various failure modes, delamination—defined as the separation of layers within the composite structure—stands out as the most common and critical type of failure in these materials. In this study, the mode I interlaminar fracture energy was predicted using the virtual crack closure technique (VCCT) integrated with a finite element model (FEM), applied to a double cantilever beam (DCB) specimen. Additionally, a straightforward analytical model was developed to calculate the critical fracture energy in mode I. The analytical model used the material strength and stiffness. The results demonstrated strong agreement with experimental data, with a margin of error as low as 5%, highlighting the accuracy and reliability of the proposed methods.

## 1. Introduction

Composite laminates are strong challenges to many traditional metals like steel and aluminium due to their excellent specific strength, light weight, and high resistance to corrosion and erosion. Delamination, which is defined as the separation of layers, is considered the primary and most serious failure mode in these materials. Therefore, significant effort must be assigned to understanding and predicting this phenomenon. In recent years, various methods and models have been proposed to predict delamination growth [1,2] and transverse cracking [3,4,5].

Many studies and approaches with different names have considered the problem of delamination, such as the cohesive fracture model [6], cohesive layer [7,8,9], and interfacial decohesion [8]. The virtual crack closure technique (VCCT) [10] is widely recognized as the most effective tool to approximate the surface release energy G. This method requires determination of the nodal forces at the delamination tip, the displacement openings directly behind it, and the virtual area in front of the crack tip. To calculate the release energy G for the mixed-mode in a general case, the nodal forces and the displacement openings must be transformed into an instantaneous local coordinate system aligned with the delamination tip, which is only possible after determining the normal direction over delamination path [11].

The delamination usually gets measured using a double cantilever beam (DCB) specimen [12,13]. The finite element method was widely used for the simulation of delamination failure using surface-based cohesive [11], cohesive element [14,15], or even virtual crack closure techniques [16,17]. Delamination failure is important both when the laminated composite is in service and through machining or cutting like drilling [13]. In a model by Li [18], delamination and transverse crack growth in laminated composite panels and shells was predicted. It considered interlaminar and intralaminar damage mechanisms using fracture mechanics and cohesive zone modelling. The approach considered material properties, loading conditions, and geometric factors that influence crack propagation. Numerical simulations validated the model against experimental data and proved its accuracy. The results improved the understanding of composite failure and helped in the design of more durable structures. Whitcomb [19] investigated delamination growth in composite plates due to local buckling using a 3D nonlinear finite element model and a strain energy release rate-based fracture criterion. Chen [20] examined elastic buckling and post-buckling behaviors in an axially loaded beam plate with transverse delamination, employing shear deformation theory and a Griffith-type fracture criterion, confirming the reduction in critical buckling and ultimate loads due to shear deformation. Hitchings et al. [21] introduced a finite element technique for modelling arbitrarily shaped delamination growth in laminated composites. They combined linear elastic fracture mechanics (LEFM) and a strain energy release rate-based criterion to be applied along the delamination tip. Aymerich et al. [22] employed the virtual internal bond model to predict delamination initiation and growth in unidirectional laminated composites, including DCB, end notch flexure (ENF), and mixed-mode bending (MMB) tests. The staking sequences layup geometry was used to calculate the fiber tension surface release energy [23], and a simple analytical model was extracted, which had a high degree of accuracy. Additionally, researchers attempted to calculate the fracture properties using non-traditional testing operations for composite laminates using natural frequencies of strikes on a DCB [24]. Meer et al. [25] proposed a method for progressive delamination modelling using a level set field to implicitly define the crack front, introducing weak discontinuities to capture transitions between cracked and uncracked regions, and applied an explicit energy-based relation where crack growth was determined by the configurational force derived from other mathematical models.

As mentioned before, there is a great need for a deeper understanding of the failure modes of composite laminates, so the present study deals with three main objectives: (1) development of a Virtual Crack Closure technique in combination with FEM to simulate delamination failure, (2) analytical prediction of the resistance curve using the VCCT and the presented equations, and (3) prediction of the failure load after reaching the peak load, where only the material strength and the beam geometry are known.

The work was structured as follows: In Section 2, the VCCT was outlined, then the analytical model was extracted in Section 3, followed by Section 4, in which the corresponding finite element model was extracted. In Section 5, the results were correlated and then the conclusions and adjustments were outlined.

## 2. Virtual Crack Closure Technique (VCCT)

**Figure 1:**
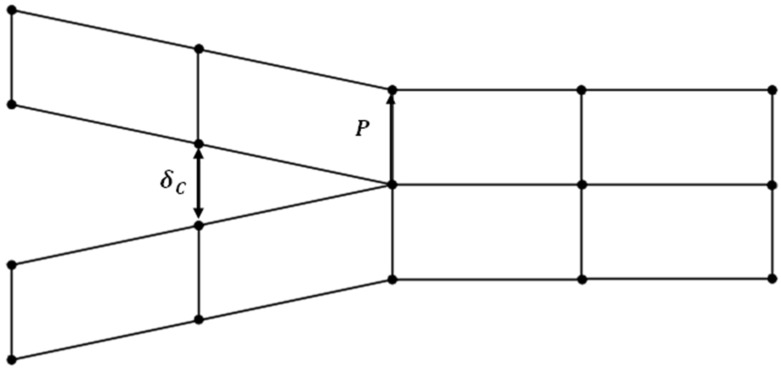
Nodal reaction force (P) and critical separation $\delta_{Cr}$ used in Equation (1) of crack tip [27].

The basic idea of the (VCCT), a popular technique for calculating the energy release rate and modelling crack propagation in fracture mechanics, is depicted in the drawing in Figure 1. It specifically shows the separation at a virtually opened fracture tip and the nodal response forces, which are crucial components in assessing the stress intensity parameters. In the situation of VCCT, the technique assumes that the energy required to close a virtually opened crack equals the energy needed to propagate the crack further. This assumption is modelled by computing the forces acting on the crack faces (nodal reaction forces) and the corresponding displacements or separations at these nodes. The interaction between the nodal forces and separations provides a direct estimation of the strain energy release rate, as proposed by Rybicki and Kanninen in their work on VCCT [10]. The relative displacements between neighboring nodes at the fracture tip brought on by external loading are represented by the crack face separation, which was depicted in Figure 1. Finding the crack-opening mode (Mode I), shear mode (Mode II), or tearing mode (Mode III), which together characterize the fracture mechanics behavior of the structure being studied, requires these divisions. In a similar manner, the internal stresses that prevent the crack faces from virtually separating are represented by the nodal response forces, which balance the external loading conditions. The VCCT framework, which combines these parameters, offers a computationally efficient and reliable method for predicting the onset and propagation of cracks in a variety of materials and structures. For applications in energy systems, mechanical engineering, and aerospace where the integrity of materials under load is crucial, this makes it especially helpful [26].

### 2.1. Damage Evaluation Criteria

**Figure 2:**
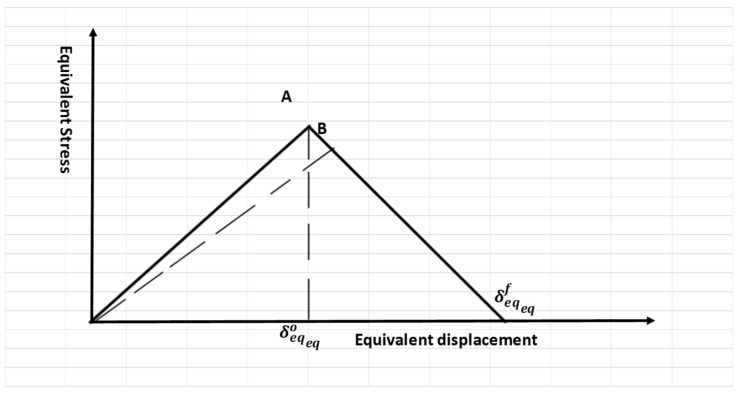
Linear damage law.

Delamination failure involves crack initiation and crack growth. These two mechanisms are governed by the principles of damage development. When the load reaches a critical value or the maximum peak load, failure occurs. This initiation criterion was established by linear evaluation laws. These evaluation laws reduce the need for refining mesh sizes [28,29,30]. A typical linear tensile separation response is shown in Figure 2. The onset of damage is represented by the initial slope, followed by softening, characterized by the negative linear portion of the curve, which indicates failure beyond the elastic region.

The variable D can be calculated as follows:

(1) $$D=\frac{\delta_{eq}^{f}\left(\delta_{eq}-\delta_{eq}^{o}\right)}{\delta_{eq}\left(\delta_{eq}^{f}-\delta_{eq}^{o}\right)}$$

where D damage variable factor, $\delta_{eq}^{o}$, and $\delta_{eq}^{f}$ are the equivalent displacements at the end of initiation and the end of failure, respectively.

### 2.2. Finite Element Modelling

**Figure 3:**
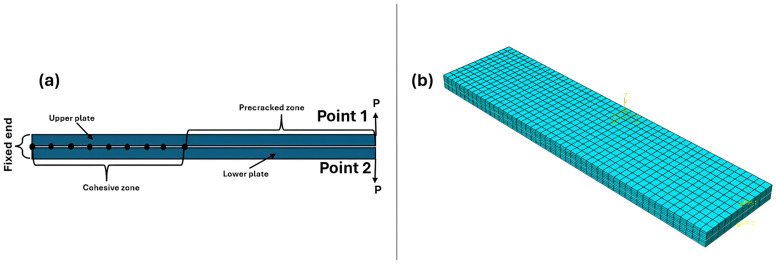
FEM: (**a**) boundary conditions and (**b**) mesh domain.

The DCB test was simulated using two plates with 24.4 mm width, 101.6 mm length, 2.14 mm thickness, and pre-crack length 50.8 mm (see Figure 3a). The model was created as a pair of contact with interaction properties for VCCT as mixed-mode (BK) with maximum tangential stress, and the interfacial mixed model values are listed in Table 1. The surface based cohesive zone (SCZM) uses the same interaction behaviors previously discussed, but the cohesive penalty interfacial stiffness is equal to ($K_{o}=4\times 10^{5}$ N/mm^3^) [31]. The maximum traction separation N = 80 MPa [31]. This method was used to validate the VCCT as previously explained. The implemented initial clearance was $1\times 10^{-5}$. This method was completely described in Abdellah [11]. The linear evaluation law for both methods were linear shapes, as illustrated in Figure 2. A bilinear, four-node, plane stress quadrilateral element (CPS4R) with reduced integration and deletion capability was employed in the finite element analysis. This element offers a good compatibility between computational efficiency and accuracy, and reduced integration element type in numerical stiffness and hypothetical hourglassing improves performance for large-scale simulations. The deletion feature allows for realistic modelling of material failure, crucial for studying crack initiation and propagation. The mesh, detailed in Figure 3b, utilized 1 mm elements with 2244 elements. The load (P) was applied to point 1, and 2, which coupled with the top and bottom surface of the DCB, as shown In Figure 3a. In previous studies [32,33], the mesh convergence was establish to have little effect in the case of measuring the load carrying capacity of composite plate. Additionally, the linear evaluation damage model in elastic materials reduces sensitivity to mesh density.

## 3. Analytical Model

### Delamination

Delamination is the process by which layers in a laminated composite material separate from one another. The load carrying capability of the entire laminate may have been impacted by impact, manufacturing flaws, and cyclic loading. This type of failure typically occurs in applications where composite laminates are widely employed, such as aerospace, automotive, and wind energy applications. Many numerical and experimental studies provide a good understanding of delamination in laminated composite materials [35]. However, further studies are needed to optimize and deepen the understanding of the failure modes in these materials.

The resistance of delamination measured by the interlaminar fracture energy $G_{IC}$ in mode I

(2) $$G_{IC}=\frac{3P\delta_{C}}{2B\left(a+\left|∆\right|\right)}$$

where *P* is load, $\delta_{C}$ is the critical displacement (computed using VCCT), *B* is the specimen width, *a* is the initial delamination length, and $\left|∆\right|$ is the absolute correction factor determined form the relationship between compliance and delamination length, which would be predicted using VCCT. Compliance relation according to beam theory can be measured as follows:

(3) $$C^{1/3}=m\left(a+\left|∆\right|\right)$$

where m is the linear regression slope.

**Figure 4:**
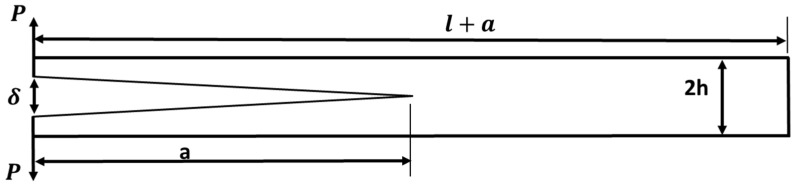
Problem of double cantilever beam.

In the pre-cracked DCB shown in Figure 4, the arms can be considered like a cantilever beam supported at the right end and loaded at the lift end. The model was based on beam theory [36], and the beam deflection δ (COD) can be calculated using Equation (4) for one DCB arm as follows:

(4) $$\delta =\frac{P\times a^{3}}{3\;EI}$$

where *I* is the second moment of inertia, P is the load, and a is the pre-crack length. Additionally, considering the deflection for both DCB arms, and substitution by the value of moment of inertia for rectangular geometry ($I=\frac{Bh^{3}}{12}$), the following Equation (5) can be used for total deflection:

(5) $$\delta_{Cr}=\frac{4Pa^{3}}{\;EBh^{3}}$$

The compliance of specimen Equation (3), therefore it can be rewritten with respect to applied load and beam stiffness as follows:

(6) $$C=\frac{8a^{3}}{EBh^{3}}$$

The surface release energy $G_{IC}$ as stated in Equation (2) related to compliance, therefore it can be rewritten as follows:

(7) $$G_{IC}=\frac{P^{2}\partial C}{2\partial A}$$

where ∂A is the crack extension area, and the h is the arm thickness.

By applying the differentiation for compliance Equation (6) and implemented through Equation (6), the surface release energy $G_{IC}$ can be calculated as follows:

(8) $$G_{IC}=\frac{{12P}^{2}a^{2}}{EB^{2}h^{3}}$$

The arms of the cantilever beam shown in Figure 4 were subjected to bending moment; therefore, the bending stress can be calculated as follows:

(9) $$M=\frac{\sigma_{b}\;I}{h}$$

Based on beam theory and linear elastic fracture mechanics fundamentals, the surface release energy can be calculated using the work done and energy stored through material as follows [36]:

(10) $$U_{E}=2\int_{0}^{a}\frac{M\left(x\right)}{2EI}$$

where $U_{E}$ is the elastic stored energy and $(M\left(x\right)=P\times a$) is the moment function through crack length maximum at x=a.

By substituting Equation (10) into Equation (8), considering conditions of the moment at maximum state, the surface release energy can be measured as follows:

(11) $$G_{IC}=\frac{M^{2}}{BEI}$$

Considering the following state for surface release energy in terms of COD, it can be measured as follows:

(12) $$G_{IC}={2\delta }_{Cr}\times \sigma_{b}$$

where $\sigma_{b}$ is bending stress over beam arms [11] and equal in the case of delamination testing the transverse laminate strength ${Y_{22}}^{T}$ (see Figure 4), where $\delta_{Cr}$ can be measured using VCCT previously explained for each load.

Substituting Equation (9) into Equation (11), and re-editing, the final surface release can be measured as follows:

(13) $$G_{IC}=\frac{{\sigma_{b}}^{2}\times h}{6E}$$

This equation stands alone for testing the surface release energy without the availably of COD; it just needs the specimen geometry and equivalent Young’s modulus. To calculate the peak load at which crack initiation takes place. Equating Equation (13) with Equation (8), it can be shown as follows:

(14) $$P=\frac{\sigma_{b}B\times h^{2}}{6\sqrt{2}\times a}$$

The model can not be used for multidirectional laminates or for measuring the fiber tension facture toughness because it needs the transverse tensile strength without increasing bridging of fiber, which may change the strength results. Additionally, VCCT assumes that the crack propagates in a predefined direction along the existing mesh. This can be a limitation in composite laminates where crack growth may follow complex, mixed-mode paths due to anisotropic properties. Additionally, VCCT is based on the assumption that a crack already exists and only predicts its propagation. It does not model crack initiation, which is crucial in composite laminates where damage can start as matrix cracking, fiber breakage, or delamination before an actual crack forms. This limitation makes VCCT less effective for predicting the onset of failure in composites [11,17,26].

## 4. Material

The composite laminates were unidirectional IM7/8552 carbon fiber-reinforced epoxy, and the elastic constant of such materials are listed in Table 1. These mechanical properties would be implemented in the above models. The pre-crack was an insert of 50.8 mm, which was created using 12.5 µm thick Teflon. The Teflon strips were inserted as the final step in the laminate layup. For each specimen, a straight edge was placed on the uncured laminate to mark the exact location of the intralaminar pre-crack. The specimen dimensions were 24.4 mm width, 101.6 mm length, and the specimen thickness nearly 4.34 mm. A complete description of the DCB specimens and experimental procedures would be found in Ref. [34]. The specimens were manufactured using IM7/8552 carbon fiber/epoxy pre-preg tap of 36 ply unidirectional laminates.

## 5. Results and Discussion

**Figure 5:**
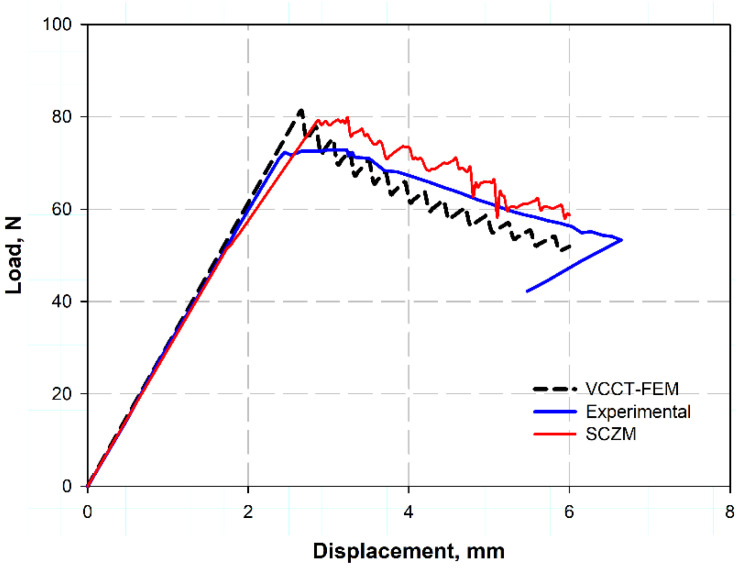
Load–displacement relation for DCB test.

Figure 5 shows the load–displacement relation for DCB measured using VCCT and SCZM compared using the experimental data found in Ref. [34]. It was clear that the prediction was highly close to the experimental data for the two models, while for VCCT it gave a more realistic trend as a zigzag shape corresponding to the loading and unloading action for the ideal test; also, it helps in measuring the compliance of the zigzag slope. Those zigzag and step-like shapes were due to stick–slip crack propagation [37,38,39], some fiber bridging in the direction of the crack, as the crack does not propagate smoothly, but rather arrests temporarily and then jumps forward when the energy release rate exceeds a critical value. Additionally, the fiber bridging the crack resists the load, therefore the load increases, and when this fiber breaks, the load suddenly decreases, making these zigzags. Additionally, due to the heterogeneity in material properties, as the carbon fiber reinforced polymer (CFRP) was an anisotropic material, they exhibit different toughness and strength through the loading direction. Moreover, it returns to frictional interaction during the fixation of the specimen or through the testing machine and other experimental results [39]. However, the predicted curve gave shorter extension than the experimental ones; this was because real CFRP laminates exhibit matrix plasticity, micro-cracking, and local damage at the crack tip. These parameters were important and serious for increasing COD, while for VCCT these factors or parameters were not included for calculations [40]. Moreover, the test machine compliance and slow crack growth in the experiment give longer and increasing COD; however, this factor were not considered in the case of VCCT [39].

**Figure 6:**
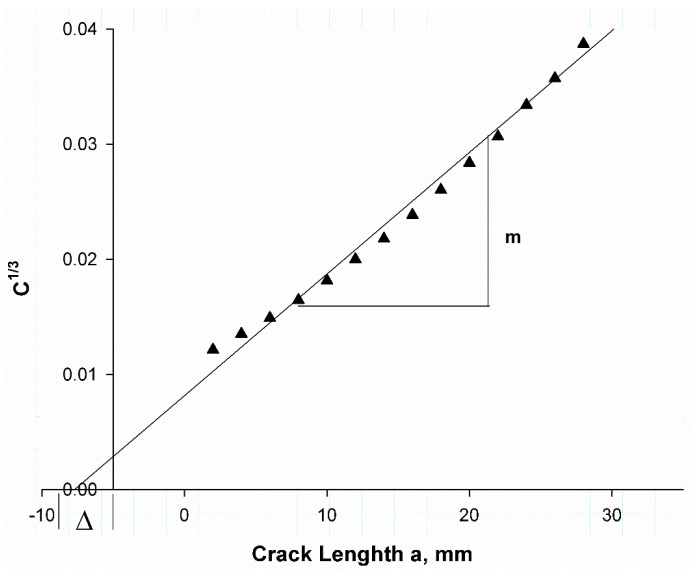
Compliance calibration fits to static data for DCB with Teflon insert.

Figure 6 shows predicted compliance using VCCT data. The compliance was calculated at each step of the curve in Figure 5 (black colored line), a crack length correction factor ∆, which can be defined as the intersect of the linear regression with the x-axis, was determined as (7.5 mm), while the experimental value was (6.57 mm) [41]. The difference was because the VCCT ignores a lot of factors in the experimental procedure such as friction between testing machine elements, interaction between layers, and some bridging in some areas during the test.

**Figure 7:**
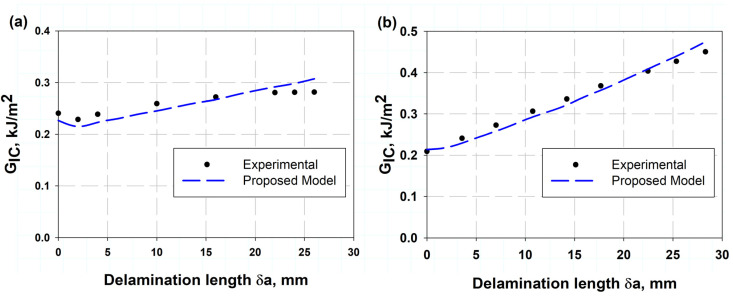
Resistance curve (R-curve) for IM7/8552 carbon fiber (**a**) with Teflon pre-crack and (**b**) with fatigue pre-crack compared with the experimental work of Ref. [34].

**Figure 8:**
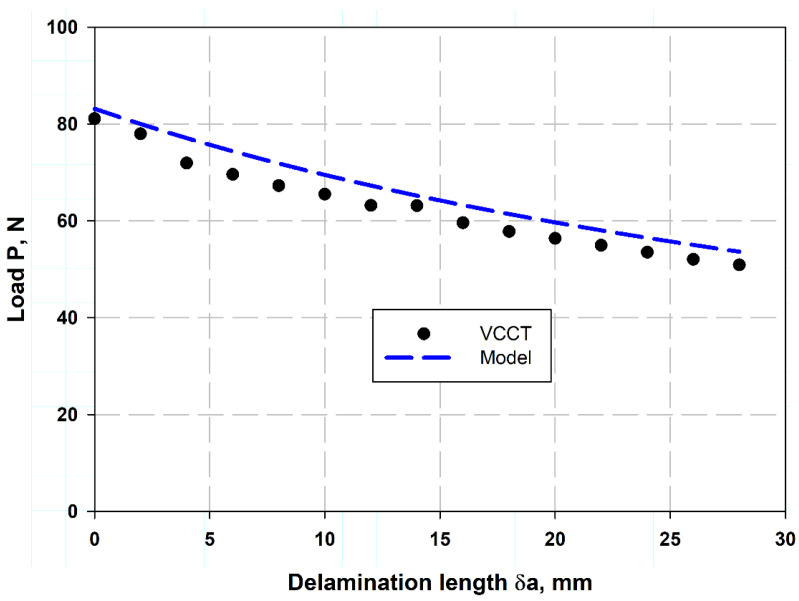
Load–delamination extension length relation.

Table 2 lists the comparison of the present model of Equation (13) with experimental data. It was observed that the percentage error was little, indicating that it was 0.77% and 1.5%, which means that the model described by Equation (13) is highly accurate, as demonstrated by the very small percentage errors when compared to experimental data. This suggests that the model can be used with confidence for predictions related to whatever it describes. Table 3 shows a comparison between the crack opening displacement (COD) values determined using the (VCCT) and the experimentally measured values [11]. The results show that the VCCT model provides a good approximation to the experimental values, with percentage errors ranging from 2% to 14%. The lowest error (2%) occurs at a COD of 3.56 mm, while the highest error (14%) is observed at 6 mm. The increasing trend in errors indicates that the VCCT model is reaching its limits by accurately predicting larger displacements, possibly due to nonlinear effects or variations in material behavior. These results are consistent with previous studies that emphasize the importance of validation in fracture mechanics simulations [42]. Figure 7a,b shows the presented VCCT model validation with the experimental results of two different sources, Czabaj and Ratcliffe [34] and Murri [41], respectively. Figure 7a illustrates the IM7/8552 carbon fiber-reinforced epoxy using the data obtain from VCCT-FEM with the help of Equation (2), considering the value of a crack length correction factor ∆=7.5 when the pre-crack length was by Teflon insert. The pre-crack was created by fatigue data in Figure 7b, and the VCCT COD value $\delta_{C}$ in Equation (2) for each load value at each slope and substituting into Equation (12). These were the two techniques, because the first technique which used the crack length correction factor misses estimating the delamination behaviors in the case of fatigue pre-crack. This can be attributed to the fatigue pre-crack being a naturally propagated crack under cyclic loading, which would give a rough, uneven crack front with some fiber bridging effects. This action introduced additional energy dissipation, whereas, for the Teflon insert pre-crack, it was perfectly sharp and clean, which would introduce a well-defined and sharp crack front. Additionally, for the case of fatigue, the softening action, residual stresses, and fiber/matrix interactions change the crack propagation characteristics [43,44]. Figure 8 shows the relation between crack length and corresponding failure mode after peak load reach, calculated using FEM associated VCCT and the analytical model presented using Equation (14). It was clearly observed that the two models give reasonably close values. The two models were validated against each other, showing good agreement. This gives confidence in the results and allows engineers to use either model for fracture toughness calculations, which are crucial for ensuring the safety and integrity of structures. The data predicted by the model obtained from Equation (13) provide reasonable results compared to the experimental work in Refs. [11,34,45]. The advantage of the present model is its simplicity and fast execution. Additionally, it can be considered a non-destructive test, requiring only the strength of the laminate in the transverse direction and the Young’s modulus in the same direction. This model can be particularly useful in material selection.

## 6. Conclusions

Delamination is one of the most critical failure modes in composite laminates. This study can be concluded into the following items:

1. Delamination is one of the most critical failure modes in composite laminates. Accurate prediction methods are essential.
2. The finite element method (FEM) has proven to be a valuable approach in predicting delamination. In this study, FEM was integrated with the VCCT.
3. The proposed model showed good agreement with experimental results:
  - IM7/8552 carbon fiber laminates: 0.77% error.
  - T300/913: 1.5% error.
4. The analytical model, developed using VCCT data, demonstrated high accuracy in predicting the resistance curve and crack opening displacement (COD).
5. Errors ranged from 2% to 14%, which is acceptable from a scientific perspective.
6. The peak load after crack initiation was estimated analytically with high accuracy.

## Figures and Tables

**Table 1:** Elastic constant of IM7/8552 carbon fiber/epoxy [34].

Properties	E1	E3 = E2	µ13	µ12	µ23	G12	G13	G23
GPa	152.69	8.703	0.32	0.32	0.45	5.16	5.16	5.16

**Table 2:** Comparison of the present model of Equation (13) with experimental data.

Laminate System	Surface Release Energy $G_{IC}$, kJ/m^2^	Model Equation (13)	Error, %
T300/913 [45]	0.258	0.26	0.77
IM7/8552 [11]	0.27	0.26	3.7
Model [11]	0.23	0.26	11
Ref. [34]	0.24	0.27	12.5

**Table 3:** VCCT (COD) and peak load predicted with analytical model and experimental work.

Experimental, δ COD, mm [11]	VCCT, δ COD, mm	Error, %
2.45	2.34	4
3.56	3.5	2
3.7	3.53	5
4.2	3.8	10
6	5.14	14

## Data Availability

The original contributions presented in this study are included in the article. Further inquiries can be directed to the corresponding author.

## References

[1] Hassan M.K., Mohammed Y., Salem T., Hashem A. (2012). Prediction of nominal strength of composite structure open hole specimen through cohesive laws. Int. J. Mech. Mech. Eng. IJMME-IJENS.

[2] Chau-Dinh T., Zi G., Lee P.-S., Rabczuk T., Song J.-H. (2012). Phantom-node method for shell models with arbitrary cracks. Comput. Struct..

[3] Nguyen-Thanh N., Valizadeh N., Nguyen M.N., Nguyen-Xuan H., Zhuang X., Areias P., Zi G., Bazilevs Y., De Lorenzis L., Rabczuk T. (2015). An extended isogeometric thin shell analysis based on Kirchhoff–Love theory. Comput. Methods Appl. Mech. Eng..

[4] Nilsson K.-F., Giannakopoulos A.E. (1995). A finite element analysis of configurational stability and finite growth of buckling driven delamination. J. Mech. Phys. Solids.

[5] Robinson P., Javidrad F., Hitchings D. (1995). Finite element modelling of delamination growth in the DCB and edge delaminated DCB specimens. Compos. Struct..

[6] Fleming D.C. (2001). Delamination Modeling of Composites for Improved Crash Analysis. J. Compos. Mater..

[7] La Saponara V., Muliana H., Haj-Ali R., Kardomateas G.A. (2002). Experimental and numerical analysis of delamination growth in double cantilever laminated beams. Eng. Fract. Mech..

[8] Camanho P.P. (2001). Numerical Simulation of Delamination Growth in Composite Materials.

[9] Roudolff F., Ousset Y. (2002). Comparison between two approaches for the simulation of delamination growth in a D.C.B. specimen. Aerosp. Sci. Technol..

[10] Rybicki E.F., Kanninen M.F. (1977). A finite element calculation of stress intensity factors by a modified crack closure integral. Eng. Fract. Mech..

[11] Abdellah M.Y. (2017). Delamination modeling of double cantilever beam of unidirectional composite laminates. J. Fail. Anal. Prev..

[12] Elder D.J., Thomson R.S., Nguyen M.Q., Scott M.L. (2004). Review of delamination predictive methods for low speed impact of composite laminates. Compos. Struct..

[13] Geng D., Liu Y., Shao Z., Lu Z., Cai J., Li X., Jiang X., Zhang D. (2019). Delamination formation, evaluation and suppression during drilling of composite laminates: A review. Compos. Struct..

[14] Lu X., Ridha M., Chen B.Y., Tan V.B.C., Tay T.E. (2019). On cohesive element parameters and delamination modelling. Eng. Fract. Mech..

[15] Kumar D., Roy R., Kweon J.-H., Choi J.-H. (2016). Numerical Modeling of Combined Matrix Cracking and Delamination in Composite Laminates Using Cohesive Elements. Appl. Compos. Mater..

[16] Marjanović M., Meschke G., Vuksanović D. (2016). A finite element model for propagating delamination in laminated composite plates based on the Virtual Crack Closure method. Compos. Struct..

[17] Krueger R., Camanho P.P., Hallett S.R. (2015). 1—The virtual crack closure technique for modeling interlaminar failure and delamination in advanced composite materials. Numerical Modelling of Failure in Advanced Composite Materials.

[18] Li D.H. (2016). Delamination and transverse crack growth prediction for laminated composite plates and shells. Comput. Struct..

[19] Whitcomb J.D. (1989). Three-Dimensional Analysis of a Postbuckled Embedded Delamination. J. Compos. Mater..

[20] Chen H.-P. (1991). Shear deformation theory for compressive delamination buckling and growth. AIAA J..

[21] Hitchings D., Robinson P., Javidrad F. (1996). A finite element model for delamination propagation in composites. Comput. Struct..

[22] Aymerich F., Lecca G., Priolo P. (2008). Modelling of delamination growth in composite laminates by the virtual internal bond method. Compos. Part A Appl. Sci. Manuf..

[23] Mohammed Y., Hassan M.K., Hashem A. (2014). Analytical model to predict multiaxial laminate fracture toughness from 0 ply fracture toughness. Polym. Eng. Sci..

[24] Abdellah M.Y., Hassan M.K., Mohamed A.F., Khalil K.A. (2021). A novel and highly effective natural vibration modal analysis to predict nominal strength of open hole glass fiber reinforced polymer composites structure. Polymers.

[25] van der Meer F.P., Moës N., Sluys L.J. (2012). A level set model for delamination—Modeling crack growth without cohesive zone or stress singularity. Eng. Fract. Mech..

[26] Krueger R. (2004). Virtual crack closure technique: History, approach, and applications. Appl. Mech. Rev..

[27] Jokinen J., Kanerva M., Wallin M., Saarela O. (2019). The simulation of a double cantilever beam test using the virtual crack closure technique with the cohesive zone modelling. Int. J. Adhes. Adhes..

[28] Abdellah M.Y., Gelany A.F., Mohamed A.F., Khoshaim A.B. (2017). Protection of limestone Coated with Different Polymeric Materials. Am. J. Mech. Eng..

[29] Abdellah M.Y. (2021). Ductile Fracture and S–N Curve Simulation of a 7075-T6 Aluminum Alloy under Static and Constant Low-Cycle Fatigue. J. Fail. Anal. Prev..

[30] Abdellah M.Y. (2017). Essential Work of Fracture Assessment for Thin Aluminium Strips Using Finite Element Analysis. Eng. Fract. Mech..

[31] Wanthal S., Schaefer J., Justusson B., Hyder I., Engelstad S., Rose C. Verification and validation process for progressive damage and failure analysis methods in the NASA Advanced Composites Consortium. Proceedings of the American Society for Composites (ASC) Technical Conference.

[32] Alharthi H., Abdellah M.Y. (2025). Stress Analysis and Strength Prediction of Carbon Fiber Composite Laminates with Multiple Holes Using Cohesive Zone Models. Polymers.

[33] Alssayegh A., Abdellah M.Y., Hassan M.K., Azam S., Melaibari A., Khashaba U. (2025). Optimizing high cycle fatigue predictions in notched Al 7075-T6: An analytical approach to rotating bending behavior. Results Eng..

[34] Czabaj M.W., Ratcliffe J.G. (2013). Comparison of intralaminar and interlaminar mode I fracture toughnesses of a unidirectional IM7/8552 carbon/epoxy composite. Compos. Sci. Technol..

[35] Librescu L., Song O. (2005). Thin-Walled Composite Beams: Theory and Application.

[36] Wang C.H. (1996). Introduction to Fracture Mechanics.

[37] Song W., Chen Y., Mu Z., Wang Y., Zhang Z., Wang Z., Liu L., Zhang B., Li Y., Li B. (2022). A feather-inspired interleaf for enhanced interlaminar fracture toughness of carbon fiber reinforced polymer composites. Compos. Part B Eng..

[38] Dávila C.G., Rose C.A., Camanho P.P. (2009). A procedure for superposing linear cohesive laws to represent multiple damage mechanisms in the fracture of composites. Int. J. Fract..

[39] Budiansky B., Hutchinson J.W., Evans A.G. (1986). Matrix fracture in fiber-reinforced ceramics. J. Mech. Phys. Solids.

[40] Reeder J.R., Crews J.H. (1990). Mixed-mode bending method for delamination testing. AIAA J..

[41] Murri G.B. Evaluation of delamination growth characterization methods under mode I fatigue loading. Proceedings of the 15th US-Japan Conference on Composite Materials.

[42] Anderson T.L., Anderson T.L. (2005). Fracture Mechanics: Fundamentals and Applications.

[43] Khan R. (2019). Fiber bridging in composite laminates: A literature review. Compos. Struct..

[44] Krueger R. (2008). An Approach to Assess Delamination Propagation Simulation Capabilities in Commercial Finite Element Codes.

[45] Pinho S.T. (2005). Modelling Failure of Laminated Composites Using Physically-Based Failure Models.

